# Disproportion and Decision: Ethnic Minority Overrepresentation and Police Risk Assessment in Missing Persons Cases

**DOI:** 10.3390/bs15121628

**Published:** 2025-11-27

**Authors:** Fiona Gabbert, Adrian J. Scott, Karen Shalev, Amy van Langeraad

**Affiliations:** 1 School of Mind, Body and Society, Goldsmiths, University of London, London SE14 6NW, UK; a.scott@gold.ac.uk (A.J.S.); a.vanlangeraad@gold.ac.uk (A.v.L.); 2 School of Criminology and Criminal Justice, University of Portsmouth, Portsmouth PO1 2UP, UK; karen.shalev@port.ac.uk

**Keywords:** missing persons, policing, risk assessment, ethnicity, disproportionality, investigative bias, cultural competence

## Abstract

Disproportionality in missing persons cases raises critical questions about forensic and legal decision making. In the UK, Black individuals comprise 14% of missing persons but only 3% of the population. This study analysed 18,266 cases from nine police forces in England and Wales to examine how case characteristics and ethnicity influence risk assessments. Analyses proceeded in three stages: (i) descriptive profiling of demographic, contextual, and risk-related factors; (ii) statistical comparison across ethnic groups; (iii) predictive modelling of how these characteristics influence risk classification. Ethnicity did not independently predict risk classification once other characteristics were controlled for. However, characteristics disproportionately associated with Black missing persons, such as youth and care orders, were linked to lower risk classifications. In contrast, White individuals were more often reported with mental health, health, or harm risks, which strongly predicted high-risk classification. This suggests police decision making may be indirectly shaped by ethnicity via associated characteristics, raising concerns about equity in assessment and investigative prioritisation. Potential mechanisms include underreporting of vulnerabilities in minority communities and inconsistencies in police recording practices. The study highlights the need for culturally informed, evidence-based decision frameworks in missing persons investigations to support just and accurate decision making in policing.

## 1. Introduction

A missing person is formally defined as “anyone whose whereabouts cannot be established … and their well-being or otherwise confirmed” (College of Policing, 2021). While simple in definition, the concept encompasses a complex social and institutional challenge with wide-ranging implications for individuals, families, communities, and the police. The scale of the problem in the United Kingdom is substantial; during 2023/2024, police forces in England and Wales recorded 378,272 missing person related calls (UK Missing Persons Unit, 2024). These figures underscore the reality that missing persons are not a marginal phenomenon but a recurring and resource-intensive issue that shapes the day-to-day functioning of policing.

The consequences of missing person cases are multiple and layered. Families of those who go missing experience considerable distress, uncertainty, and disruption, often reporting emotional trauma that persists long after the case is resolved (Parr & Fyfe, 2013). For policing organisations, the cumulative workload involved in responding to missing person cases is immense. Shalev-Greene and Pakes (2014) estimate that a single medium-risk investigation can cost up to £2500, while the aggregate time spent annually equates to the work of more than 19,000 full-time officers. Such figures not only highlight the financial costs but also indicate the opportunity costs associated with missing person investigations, as resources devoted to these cases inevitably reduce capacity elsewhere. Importantly, as annual reports from the National Crime Agency (NCA) demonstrate, the number of recorded missing person cases continues to rise year-on-year. It is therefore reasonable to assume that both the direct financial costs and broader opportunity costs have grown since these estimates were first calculated.

Given finite resources, decision making becomes central to missing person investigations. Risk assessment has become a cornerstone of operational practice, providing the primary mechanism by which decisions about resource allocation are structured. At the time of this study, the College of Policing (2021)^1^ defined four categories of risk: no apparent risk (where the risk of harm is considered absent), low risk (where the possibility of harm exists but is minimal), medium risk (where harm is likely but not expected to be serious), and high risk (where serious harm is considered very likely). These categories are not merely descriptive but serve as decision-making tools that shape investigative trajectories. Cases assessed as higher risk mobilise more resources, while those deemed lower risk may receive reduced attention. Notably, the majority of missing person cases in the UK are classified as medium risk (UK Missing Persons Unit, 2021). This category obliges the police to undertake active searching and to provide ongoing support to those reporting the disappearance. As such, the accuracy, consistency, and fairness of decision making during risk assessment is pivotal, both for investigative effectiveness and for public trust in policing.

Despite the existence of structured frameworks, concerns have emerged about the fairness and consistency of decision making in missing person investigations. One particularly troubling issue is the disproportionate representation of ethnic minority groups within the missing population. In 2019/20, 23 police forces across England and Wales reported that Black individuals were overrepresented among missing persons relative to their proportion in the general population (UK Missing Persons Unit, 2021). During this period, Black individuals accounted for 14% of recorded missing cases despite comprising only 3% of the overall population (UK Government, 2020). Such disparities raise questions not only about broader social inequalities but also about whether investigative decisions, particularly risk classifications, are applied equitably across groups.

Emerging evidence also points to differential outcomes once individuals are reported missing. Archival analyses indicate that White missing persons are statistically more likely to be located than non-White missing persons (Hunter et al., 2023). Qualitative research supports these findings: families of Black missing persons frequently report that their concerns are dismissed or minimised, that police action is delayed or absent, and that they feel their cases are not treated with the same seriousness as others (White, 2021). These accounts suggest that decision making in case prioritisation, investigative action, and communication with families may not be applied uniformly, raising the possibility of bias, whether explicit or implicit, in the ways missing person cases are handled.

The overrepresentation of ethnic minorities within missing person populations is not unique to the UK. International studies suggest that structural inequalities in policing practices contribute to similar patterns elsewhere. In the United States, for example, concerns have long been raised about the phenomenon sometimes termed “missing white woman syndrome,” whereby disappearances of White women and girls receive disproportionate media and investigative attention compared to those of people of colour (Gilchrist, 2010; Stillman, 2022). Canadian research similarly documents inequities in the treatment of missing Indigenous women and girls, whose cases are often deprioritised or inadequately investigated (National Inquiry into Missing & Murdered Indigenous Women and Girls, 2019). These examples demonstrate that the issue is not confined to one national context but forms part of a broader global pattern of racialised disparities in criminal justice and policing. Across these contexts, the decision-making processes underpinning investigative practices are crucial to understanding how and why such disparities persist.

Within this wider literature, risk assessment practices warrant particular scrutiny. Designed to support consistent decision making, risk categorisation systems aim to reduce subjectivity and ensure resources are directed appropriately. Yet evidence from other domains of policing suggests that risk assessment is not immune to bias or inconsistency. Research on stop-and-search, bail decisions, and the use of predictive policing technologies consistently shows that race and ethnicity can shape discretionary judgments (Bowling et al., 2019; Lum & Isaac, 2016). These patterns are consistent with broader findings on implicit bias across the criminal justice system, where decisions are frequently made under conditions of time pressure, ambiguity, and incomplete information: circumstances that increase reliance on intuitive judgement and mental shortcuts (Merla et al., 2025). Under such conditions, decision-makers may unintentionally draw on automatic associations linked to race, gender, age, or socioeconomic status, influencing how cases are interpreted and prioritised even when there is a strong motivation to act impartially (Merla et al., 2025).

Empirical research shows that similar dynamics operate across forensic and legal settings, shaping sentencing, pre-trial decisions, jury deliberations, and even professional assessments by defence attorneys and forensic experts. In these contexts, social cues can subtly alter how ambiguous information is weighed, shift evaluative standards, or direct attention unevenly across cases (Merla et al., 2025). Importantly, these mechanisms do not require overt prejudice; rather, they emerge from the discretionary nature of the task and the cognitive load under which decisions are made. Given that missing-person risk assessments share many of these features (rapid judgements, limited or uncertain information, and substantial officer discretion), it is plausible that similar dynamics operate, where discretionary decision making at the point of risk assessment may inadvertently reproduce structural inequalities.

The present study seeks to contribute to this critical field of inquiry by examining missing person cases recorded by the police over a one-year period. Using administrative data provides a valuable opportunity to analyse patterns across a large number of cases, capturing demographic, contextual, and risk-related characteristics. The study pursues three primary objectives. First, it aims to describe the demographic, contextual, and risk-related features of missing person cases. Second, it examines how risk classifications are applied in practice. Third, it investigates whether these patterns vary according to the ethnicity of the missing person, thereby offering insight into potential evidence of bias in decision making during the investigative process.

As a whole, the study aims to provide an empirically grounded account of missing person investigations that advances understanding of both their operational and equity-related dimensions. In doing so, it contributes to broader debates on the role of decision making in policing, the ways in which structural inequalities may be reproduced through seemingly neutral processes, and the importance of ensuring that missing person investigations are both effective and just. Please note, this study is based on the same dataset as an earlier technical report produced for the Home Office, and some overlap in methodological description is therefore unavoidable. The current manuscript provides a revised and extended academic analysis beyond the scope of the original report.

## 2. Materials and Methods

### 2.1. Sample

Police forces in England and Wales that use the Community Policing and Case Tracking (COMPACT) system to record missing person cases were invited to take part in the research. COMPACT is a specialist software used by many UK police forces to manage and share details related to missing person cases, including personal information and potential risk factors. The invitation explained the purpose of the research and confirmed that information collected during the course of the research would be kept strictly confidential, and that data would not be attached to any individual or police force in an identifiable way. Nine police forces (out of 43 forces in England and Wales) indicated that they were eligible and willing to participate. They were subsequently sent a code and instructions to extract one year’s worth of data relating to missing person cases held on COMPACT. The dataset was emailed securely to a Police Service Volunteer (PSV) who managed a team of other PSVs who redacted any details that could be used to identify specific individuals. The anonymised datasets were then emailed to the Research Team.

### 2.2. Quantitative Data

One year’s worth of data from nine UK (England and Wales) police forces regarding all missing person cases recorded using COMPACT case management software between 1 January 2021 and 31 December 2021 were shared with the Research Team (see Appendix A for the data fields requested from COMPACT). These data were combined to form a single dataset for the purpose of statistical analysis and cleaned to remove incomplete and repeat missing person data. The initial sample comprised 45,750 missing person cases and the final sample comprised 18,266 missing person cases.

Incomplete data for age, sex, risk, publicity, and harm were removed from the dataset given the small numbers involved. However, this strategy was not appropriate for nationality, marital status, and sexuality given the large amount of incomplete data. Therefore, we retained the cases with incomplete data and excluded these three characteristics from the subsequent statistical analyses. Regarding ethnicity, cases were removed from the dataset if, (i) there were incomplete or ambiguous (e.g., ‘British’) data for both data fields (ethnic appearance and ethnicity), (ii) there were inconsistencies in categorisation across the two data fields (e.g., ethnic appearance = White, ethnicity = Mixed/other), and (iii) it was not possible to gather information about the missing person’s ethnicity elsewhere in the dataset. Furthermore, we retained only the first recorded case (i.e., the earliest by date) for any repeat missing person data. This approach focused the analysis on unique missing persons rather than the total number of missing incidents, as some individuals went missing multiple times (ranging from 2 to 112 instances). Including repeat cases could have skewed the sample and affected the reliability of the statistical analysis. By using only the first case per person, we reduced this potential bias.

### 2.3. Qualitative Data

Five PSVs worked to redact any identifying details from free-text data fields in the COMPACT database. These fields contained information like the circumstances of disappearances and suspected reasons for going missing. The redaction process removed over 386,000 pieces of personal information (e.g., names and addresses).

Two specific free-text data fields, warning signals and person risk factor flags, were analysed using a deductive coding framework. This framework was based on the 19 initial risk factor categories in COMPACT. These fields were coded to identify whether each risk factor was present or absent, ensuring a thorough recording of risk factors. For example, the ‘warning signals’ field sometimes mentioned conditions like anxiety or depression, even when the corresponding risk factor field in the database did not explicitly indicate a mental health issue. This process allowed us to better align our risk groups with the key ‘reasons for missing incidents’ described in the Missing Persons Data Report 2019/20 (National Crime Agency, 2021; see Appendix B for the deductive coding framework groups for the 19 initial risk factor categories).

## 3. Results

### 3.1. Key Characteristics

The average age of missing persons was 28.72 years (*SD* = 19.02). 10,681 (58.5%) of missing persons were male, 7523 (41.2%) were female, and 62 (0.3%) were transgender. Regarding ethnicity, 15,074 (82.5%) were White, 1549 (8.5%) were Black, 1211 (6.6%) were Asian, and 432 (2.4%) were of a Mixed/other ethnicity.^2^ A total of 3712 (20.3%) of missing persons were reported as having a disability, and 3970 (21.7%) were reported as having a mental illness. A total of 1083 (5.9%) of missing persons had an absconder order (i.e., they have left or escaped from lawful custody), such as before or after a conviction, and 2016 (11.0%) had a care order (i.e., a child has been taken into care by a local authority). Regarding the location they went missing from, 14,180 (77.6%) were last seen in their home or neighbourhood, and 4086 (22.4%) were last seen in another location (e.g., a care home).

A total of 12,858 (70.4%) missing persons were reported as having a vulnerability risk, 12,660, (69.3%) were reported as having a personal circumstances risk, 11,273 (61.7%) were reported as having a health risk, 7244 (39.7%) were reported as having a harm risk, and 3397 (18.6%) were reported as having an ‘other’ risk. This other risk category captured vulnerability indicators that did not fit predefined fields. In UK policing guidance, these commonly include circumstantial or situational risks such as recent traumatic events, domestic abuse concerns, threats from others, pregnancy, or other contextual information suggesting heightened danger (College of Policing, 2021). Finally, 3509 (19.2%) of missing persons were allocated high risk, 12,852 (70.4%) were allocated medium risk, 1718 (9.4%) were allocated low risk, and 187 (1.0%) were allocated no apparent risk.

### 3.2. Ethnicity

We examined whether there are any statistically significant differences in the key characteristics of interest in missing person cases according to the ethnicity of the missing person. A Bonferroni-corrected alpha value of 0.004 was applied to protect against Type I errors. These comprised the age and sex of the missing person, the location they went missing from; whether they were recorded as having a disability and/or mental illness; whether they had an absconder and/or care order; the five risk factor groups—vulnerability risk, personal circumstances risk, health risk, harm risk, and other risk; and the level of risk allocated to each missing person case.

A one-way ANOVA was performed to compare the effect of the missing person’s ethnicity on age. There was a statistically significant difference between groups, *F*(3, 18,262) = 81.58, *p* < 0.001. A Tukey post hoc test revealed that White missing persons (*M* = 29.67, *SD* = 19.43) were significantly older than the other three ethnicity groups. In addition, Asian (*M* = 25.64 years, *SD* = 16.35) and Black (*M* = 24.26 years, *SD* = 17.13) missing persons were significantly older than missing persons of a Mixed/other ethnicity. A series of chi-square analyses were performed for the remaining key characteristics and statistically significant differences were observed between ethnicity and several characteristics (see Table 1).

In brief, White missing persons were more likely to be female than those of Black and Mixed/other ethnicities, whilst Black and Mixed/other missing persons were more likely to be male than those of White ethnicity. Asian, Black and White missing persons were more likely to have a recorded disability than those of Mixed/other ethnicity. White missing persons were more likely to have a recorded mental illness than those of Black and Mixed/other ethnicities, whilst Black and Asian missing persons were more likely to have a recorded mental illness than those of Mixed/other ethnicity. Black missing persons were also more likely to be subject to a care order than those of White and Asian ethnicity, and White missing persons were more likely to have a care order than those of Asian ethnicity. Differences were also found in the locations missing persons went missing from, whereby Asian and White missing persons were more likely to be last seen in their home or neighbourhood than those of Black and Mixed/other ethnicities.

Regarding risk factors, Black missing persons were more likely to have an identified vulnerability risk than those of White and Asian ethnicities. White missing persons were more likely to have a recorded health risk or harm risk than all other ethnicities. Regarding level of risk, White missing persons were more likely to be allocated high risk than all other ethnicities. Mixed/other missing persons were more likely to be allocated medium risk than those of Asian and White ethnicities, whilst Black missing persons were more likely to be allocated medium risk than those of White ethnicity. Finally, Asian missing persons were more likely to be allocated low risk than those of Mixed/other ethnicity. Collectively, these findings show statistically significant differences in key characteristics according to the ethnicity of the missing person.

### 3.3. Risk Classification

We subsequently performed three purposeful logistic regression analyses, using Hosmer et al.’s (2013) seven step model building process, to examine the relationship between the key characteristics of interest and the risk classification: (i) low vs. medium risk, (ii) low vs. high risk, and (iii) medium vs. high risk. Due to the very small number of cases in the no apparent risk group (*n* = 187), we incorporated these into the low-risk category. It is also important to note that it was not possible to include transgender individuals (*n* = 62) in the analyses because of insufficient data. Therefore, the key characteristics comprised age (years), sex (female, male), ethnicity (Black, Asian, Mixed/other, White), and location missing from (home or neighbourhood, other), as well as disability (no/unknown, yes), mental illness (no/unknown, yes), absconder order (no/unknown, yes), care order (no, yes), vulnerability risk (no/unknown, yes), personal circumstances risk (no/unknown, yes), health risk (no/unknown, yes), harm risk (no/unknown, yes), and other risk (no/unknown, yes).

The seven steps comprised: (1) performing analyses of variance and chi-square analyses to identify key characteristics with *p*-values less than 0.25; (2) entering these characteristics into initial models and removing non-significant characteristics; (3) determining whether any removed characteristics should be re-entered into the models; (4) entering characteristics originally excluded into the models to determine whether any make significant contributions; (5) creating main effects models; (6) identifying any significant interaction terms and creating final models; (7) testing the adequacy and fit of the final models (Hosmer et al., 2013). A *p*-value of 0.25 was used during Step 1 because research suggests use of a more traditional significance level (e.g., a *p*-value of 0.05) may fail to identify important characteristics (Bendel & Afifi, 1977; Mickey & Greenland, 1989).

#### 3.3.1. Low Risk vs. Medium Risk

For low risk vs. medium risk, 11 key characteristics were included in the initial logistic regression model: age (*p* < 0.001), sex (*p* < 0.001), ethnicity (*p* = 0.005), location missing from (*p* < 0.001), absconder order (*p* < 0.001), care order (*p* < 0.001), vulnerability risk (*p* < 0.001), personal circumstances risk (*p* < 0.001), health risk (*p* = 0.061), harm risk (*p* < 0.001), and other risk (*p* < 0.001). The final model, with two non-contributing characteristics removed (ethnicity and care order), was statistically significant and correctly classified 87.1% of cases (0.7% low risk, 99.9% medium risk), χ^2^(9, *n* = 14,757) = 1369.75, *p* < 0.001. This model included nine key characteristics and explained between 8.9% (Cox & Snell R square) and 16.7% (Nagelkerke R square) of variance (see Table 2).

As shown in Table 2, a one-year *decrease* in age was associated with 1% *greater* odds of being allocated medium risk as opposed to low risk, and female missing persons were 1.13 times *more* likely to be allocated medium risk as opposed to low risk than male missing persons. Individuals who were last seen in their home or neighbourhood were 1.52 times *more* likely to be allocated medium as opposed to low risk than individuals who were last seen in another location; and missing persons with an absconder order were 1.42 times *more* likely to be allocated medium as opposed to low risk than missing persons without an absconder order. Regarding risk factors, missing persons reported as having a vulnerability risk were 4.26 times *more* likely to be allocated medium as opposed to low risk. Missing persons reported as having a personal circumstances risk were 1.17 times *more* likely to be allocated medium as opposed to low risk, and missing persons reported as having a health risk were 1.30 times *more* likely to be allocated medium as opposed to low risk. Missing persons reported as having a harm risk were 1.50 times *more* likely to be allocated medium risk as opposed to low risk, and missing persons reported as having an other risk were 1.79 times *more* likely to be allocated medium as opposed to low risk than missing persons without these respective risks.

#### 3.3.2. Low Risk vs. High Risk

For low risk vs. high risk, all 13 key characteristics were included in the initial logistic regression model: age (*p* < 0.001), sex (*p* = 0.001), ethnicity (*p* < 0.001), location missing from (*p* < 0.001), disability (*p* < 0.001), mental illness (*p* < 0.001), absconder order (*p* = 0.025), care order (*p* < 0.001), vulnerability risk (*p* < 0.001), personal circumstances risk (*p* < 0.001), health risk (*p* < 0.001), harm risk (*p* < 0.001), and other risk (*p* < 0.001). The final model, with three non-contributing characteristics removed (ethnicity, mental illness and absconder order), was statistically significant and correctly classified 75.2% of cases (51.5% low risk, 88.0% high risk), χ^2^(10, *n* = 5414) = 1430.39, *p* < 0.001. This model included 10 key characteristics and explained between 23.3% (Cox & Snell R square) and 32.1% (Nagelkerke R square) of variance (see Table 3).

As shown in Table 3, a one-year *increase* in age was associated with 1% *greater* odds of being allocated high risk as opposed to low risk, and female missing persons were 1.14 times *more* likely to be allocated high risk as opposed to low risk than male missing persons. Individuals who were last seen in their home or neighbourhood were 1.41 times *more* likely to be allocated high as opposed to low risk than individuals who were last seen in another location, and missing persons with a disability were 1.33 times *more* likely to be allocated high as opposed to low risk than missing persons without a disability. Conversely, missing persons with a care order were 2.13 times *less* likely to be allocated high as opposed to low risk than missing persons without a care order. Regarding risk factors, missing persons reported as having a vulnerability risk were 3.77 times *more* likely to be allocated high as opposed to low risk. Missing persons reported as having a personal circumstances risk were 1.97 times *more* likely to be allocated high as opposed to low risk, and missing persons reported as having a health risk were 3.42 times *more* likely to be allocated high as opposed to low risk. Missing persons reported as having a harm risk were 1.67 times *more* likely to be allocated high risk as opposed to low risk, and missing persons reported as having an other risk were 2.72 *more* likely to be allocated high as opposed to low risk than missing persons without these respective risks.

#### 3.3.3. Medium Risk vs. High Risk

Finally, for medium risk vs. high risk, all 13 characteristics of interest were included in the initial logistic regression model: age (*p* < 0.001), sex (*p* = 0.002), ethnicity (*p* < 0.001), location missing from (*p* = 0.002), disability (*p* < 0.001), mental illness (*p* < 0.001), absconder order (*p* < 0.001), care order (*p* < 0.001), vulnerability risk (*p* < 0.001), personal circumstances risk (*p* < 0.001), health risk (*p* < 0.001), harm risk (*p* = 0.053), and other risk (*p* < 0.001). The final model, with three non-contributing characteristics removed (ethnicity, location missing from, and mental illness), was statistically significant and correctly classified 79.8% of cases (97.8% medium risk, 13.7% high risk), χ^2^(10, *n* = 16,361) = 2001.69, *p* < 0.001. This model included 10 key characteristics and explained between 11.6% (Cox & Snell R square) and 17.9% (Nagelkerke R square) of variance (see Table 4).

As shown in Table 4, a one-year *increase* in age was associated with 1% *greater* odds of being allocated high risk as opposed to medium risk, and female missing persons were 1.09 times *more* likely to be allocated high risk as opposed to medium risk than male missing persons. Furthermore, missing persons with a disability were 1.33 times *more* likely to be allocated high as opposed to medium risk than missing persons without a disability. Conversely, missing persons with an absconder order were 1.35 times *less* likely to be allocated high as opposed to medium risk than missing persons without an absconder order; and missing persons with a care order were 2.17 times *less* likely to be allocated high as opposed to medium risk than missing persons without a care order. Regarding risk factors, missing persons reported as having a vulnerability risk were 1.11 times *less* likely to be allocated high as opposed to medium risk than missing persons without this risk. Conversely, missing persons reported as having a personal circumstances risk were 1.55 times *more* likely to be allocated high as opposed to medium risk. Missing persons reported as having a health risk were 2.54 times *more* likely to be allocated high as opposed to medium risk, missing persons reported as having a harm risk were 1.16 times *more* likely to be allocated high risk as opposed to medium risk, and missing persons reported as having an other risk were 1.62 times *more* likely to be allocated high as opposed to medium risk than missing persons without these respective risks.

## 4. Discussion

The purpose of this research was to gain a better understanding of why Black individuals are disproportionately represented among missing person cases in the UK. The study focused on the police decision-making processes involved in risk classification, with the hypothesis that differences in how risk is assigned to White missing persons compared with those from minority ethnic groups may contribute to this disparity. Since police risk assessments directly determine the urgency and resources allocated to locating a missing person, a lower risk classification for minority individuals may mean slower or less intensive responses, increasing the likelihood that they remain missing for longer. By examining how such decisions are made, the research aimed to assess whether these processes contribute to the ethnic disparities observed.

While ethnicity itself was not a predictor of risk classification, characteristics disproportionately associated with Black individuals were. For example, Black missing persons were younger than White missing persons and were more likely to have a care order; both of which were characteristics associated with a reduced likelihood of being allocated high risk. A different pattern emerged for White missing persons, who were more likely to have a mental illness, a health risk, and/or a harm risk than missing persons from other ethnic groups; all of which were characteristics associated with an increased likelihood of being allocated high risk.

It is possible that some of the key characteristics associated with a high-risk allocation (such as vulnerability or health concerns) are differentially (i) disclosed by the person reporting someone missing and/or (ii) recorded by the investigating officer, depending on the ethnicity of the missing person. For example, prior research highlights cultural variation in how mental health difficulties are disclosed (Loewenthal et al., 2012). Studies examining racial inequalities in mental health suggest that minority ethnic groups not only experience higher rates of such difficulties but are also disproportionately exposed to the social determinants that exacerbate them compared with White individuals. Yet these groups are often less likely to report mental health problems, frequently due to mistrust of healthcare systems (Bignall et al., 2019; Loewenthal et al., 2012). It is plausible that this reluctance extends into interactions with the police in missing person contexts. For instance, Black families may withhold information about mental health issues due to stigma, which could reduce the likelihood of a case being categorised as high risk. Distrust of policing more broadly (e.g., Office for National Statistics, 2024; Sharp & Atherton, 2007) and concerns about the potential repercussions of disclosure, such as detention under Section 136 of the Mental Health Act, or the possibility of arrest in cases where a missing individual is misidentified as an offender rather than a victim of exploitation, may also discourage the sharing of relevant vulnerabilities. Concurrently, as care orders were associated with a reduced likelihood of being allocated high risk, this finding must be interpreted within the broader context of the care system where Black children are most likely to receive the most restrictive care orders, such as secure accommodation (Edney et al., 2023). On the recording side, the current dataset does not permit assessment of whether information provided by families is consistently reflected in case records. Nonetheless, the disproportionate number of missing persons from minority ethnic backgrounds suggests that reports are indeed being made, rather than delayed or disregarded by the police.

We acknowledge a key limitation regarding the generalisability of our findings. The data analysed were from nine police forces and therefore not representative of the police service as a whole. Notably we were not able to include data from the Metropolitan Police as they do not use COMPACT to record information about missing persons. The Black population, and the missing Black population, are not evenly distributed in the general population. For example, the 2021 Census shows that 49% of Black individuals in England and Wales live in London (UK Government, 2021), and the NCA missing persons report looking at data from 2019–2020 shows that the MET accounted for 61% of all Black missing person cases (National Crime Agency, 2021). This should be considered when interpreting our findings as there is potential that different conclusions would have been drawn with a more representative sample.

A second limitation, also beyond our control, relates to the amount of incomplete and ambiguous data about a missing person’s ethnicity and/or ethnic appearance. To address our research question, it was important to have this information. However, incomplete data for ethnicity amounted to 43% of cases, while incomplete data for ethnic appearance totalled 17% of cases. Most incomplete data for ethnicity (35%) arose because the missing person had been categorised only as ‘British’ which does not convey ethnic appearance. Attempts were made to establish a person’s ethnic identity, and missing person cases were only removed if, (i) there were incomplete or ambiguous data for both variables (ethnic appearance and ethnicity), or (ii) there were inconsistencies in categorisation across the two data fields (e.g., ethnic appearance = White, ethnicity = Mixed/other). Despite this, we acknowledge a large amount of data excluded from analyses. Unfortunately, it is not possible to ascertain whether the impact of incomplete data disproportionately affected certain ethnic groups.

A final limitation relates to the recent UK Missing Persons Unit Statistical Report 2022–2023 (UK Missing Persons Unit, 2024) that revealed a lack of consistency in risk classification across police forces. For example, only 2% of missing persons were classified as high risk in Durham police force, compared to 36% in Surrey police force (Shalev & Collie, 2024). It was not possible to account for such potential disparities between forces in risk classifications in the current report.

Notwithstanding the limitations noted above, our findings point to practice improvements that may strengthen police decision making in missing person investigations. First, forces should prioritise fuller and more consistent recording of key information, particularly ethnicity, ethnic appearance, and risk-related vulnerabilities, which were frequently incomplete or ambiguously coded in our dataset. Improving data quality would reduce the likelihood that important risk-relevant information is overlooked and would support more equitable and evidence-based decision making. Second, the findings suggest a need for enhanced cultural awareness in frontline assessments. Prior research shows that some minority ethnic groups may be less willing to disclose mental health concerns (e.g., Bignall et al., 2019; Loewenthal et al., 2012), and our results indicate that high-risk factors linked to health and wellbeing were less commonly recorded for Black missing persons. Increasing officers’ awareness of cultural differences in disclosure and encouraging more active elicitation of relevant information from families, may help prevent under-identification of high-risk indicators. Together, these improvements relating to recording practice, information-gathering, and cultural competence, could help ensure that risk assessments are both more accurate and more consistent across demographic groups.

## 5. Conclusions

Characteristics associated with lower risk classification (e.g., care orders, vulnerability risks, and last seen in locations other than home) were more common among Black individuals in the sample. Conversely, White individuals were more likely to exhibit characteristics linked to higher overall risk classification, such as mental illness or health and wellbeing risks. Thus, although ethnicity alone did not explain the differences in risk allocation, these factors go some way to explaining why Black individuals were less often rated as high risk and more often rated as medium risk.

Two key recommendations relating to training have arisen from this research. First, for police officers to record information in full on the COMPACT (or equivalent) system, especially relating to a missing person’s ethnicity and ethnic appearance, and to avoid ambiguous terms (such as ‘British’). Second, for police officers to be more culturally aware of potential reluctance to disclose certain information about a missing person (e.g., mental health concerns). We know from the available data that certain vulnerability, personal, health, harm, and other risks inform the risk classification. Thus, it is possible that some minority ethnic groups are being disadvantaged by police officers not seeking full information about the missing person.

## Figures and Tables

**Table 1:** Frequencies and Chi-Square Results for Key Characteristics According to Ethnicity.

Key Characteristic	Black		Asian		Mixed/Other		White		χ^2^	df	*p*
	n	%	n	%	n	%	n	%			
Sex									42.64	6	<0.001
Female	543	35.1	474	39.1	152	35.2	6354	42.2			
Male	1004	64.8	732	60.4	277	64.1	8668	57.5			
Transgender	2	0.1	5	0.4	3	0.7	52	0.3			
Disability									40.19	3	<0.001
Yes	322	20.8	284	23.5	40	9.3	3066	20.3			
No/unknown	1227	79.2	927	76.5	392	90.7	12,008	79.7			
Mental illness									50.41	3	<0.001
Yes	289	18.7	267	22.0	41	9.5	3373	22.4			
No/unknown	1260	81.3	944	78.0	391	90.5	11,701	77.6			
Absconder order									8.92	3	0.030
Yes	106	6.8	51	4.2	24	5.6	902	6.0			
No/unknown	1443	93.2	1160	95.8	408	94.4	14,172	94.0			
Care order									24.74	3	<0.001
Yes	210	13.6	92	7.6	50	11.6	1664	11.0			
No	1339	86.4	1119	92.4	382	88.4	13,410	89.0			
Location missing from									49.79	3	<0.001
Home or neighbourhood	1154	74.5	983	81.2	287	66.4	11,756	78.0			
Another location	395	25.5	228	18.8	145	33.6	3318	22.2			
Vulnerability risk									26.27	3	<0.001
Yes	1172	75.7	829	68.5	317	73.4	10,540	69.9			
No/unknown	377	24.3	382	31.5	115	26.6	4534	30.1			
Personal circumstances risk									4.10	3	0.251
Yes	1051	67.9	853	70.4	287	66.4	10,469	69.5			
No/unknown	498	32.1	358	29.6	145	33.6	4605	30.5			
Health risk									443.84	3	<0.001
Yes	725	46.8	549	45.3	174	40.3	9825	65.2			
No/unknown	824	53.2	662	54.7	258	59.7	5249	34.8			
Harm risk									35.03	3	<0.001
Yes	520	33.6	445	36.7	160	37.0	6119	40.6			
No/unknown	1029	66.4	766	63.3	272	63.0	8955	59.4			
Other risk									4.52	3	0.210
Yes	307	19.8	218	18.0	93	21.5	2779	18.4			
No/unknown	1242	80.2	993	82.0	339	78.5	12,295	81.6			
Risk level									87.45	9	<0.001
High	214	13.8	183	15.1	55	12.7	3057	20.3			
Medium	1174	75.8	870	71.8	345	79.9	10,463	69.4			
Low	138	8.9	141	11.6	25	5.8	1414	9.4			
No apparent risk	23	1.5	17	1.4	7	1.6	140	0.9			

Note. A Bonferroni-corrected alpha value of 0.004 was applied to protect against Type I errors.

**Table 2:** Initial and Final Logistic Regression Models Predicting Level of Risk (Low vs. Medium).

Key Characteristic	Initial Model					Final Model				
	*B*	*SE*	*p*	*OR*	95% CI	*B*	*SE*	*p*	*OR*	95% CI
Age	−0.02	0.00	<0.001	0.98	[0.98, 0.99]	−0.16	0.00	<0.001	0.98	[0.98, 0.99]
Sex	0.12	0.06	0.024	1.13	[1.02, 1.26]	0.12	0.06	0.026	1.13	[1.02, 1.26]
Ethnicity			0.081					-		
Asian	−0.15	0.13	0.234	0.86	[0.67, 1.10]	-	-	-	-	-
Mixed/other	0.37	0.21	0.078	1.45	[0.96, 2.20]	-	-	-	-	-
White	0.02	0.09	0.853	1.02	[0.85, 1.22]	-	-	-	-	-
Location missing from	0.41	0.07	<0.001	1.51	[1.32, 1.74]	0.42	0.07	<0.001	1.52	[1.32, 1.75]
Absconder order	0.36	0.15	0.013	1.44	[1.08, 1.91]	0.35	0.14	0.015	1.42	[1.07, 1.88]
Care order	−0.06	0.10	0.288	0.95	[0.77, 1.16]	-	-	-	-	-
Vulnerability risk	1.45	0.06	<0.001	4.27	[3.84, 4.76]	1.45	0.05	<0.001	4.26	[3.84, 4.74]
Personal circumstances risk	0.16	0.06	0.004	1.17	[1.05, 1.30]	0.16	0.06	0.005	1.17	[1.05, 1.30]
Health risk	0.25	0.06	<0.001	1.29	[1.15, 1.44]	0.26	0.06	<0.001	1.30	[1.16, 1.45]
Harm risk	0.41	0.06	<0.001	1.50	[1.34, 1.69]	0.41	0.06	<0.001	1.50	[1.34, 1.68]
Other risk	0.58	0.09	<0.001	1.79	[1.51, 2.12]	0.58	0.09	<0.001	1.79	[1.51, 2.11]

Note. Reference categories: Sex = male, Ethnicity = Black, Location missing from = other, Absconder order = no/unknown, Care order = no, Vulnerability risk = no/unknown, Personal circumstances risk = no/unknown, Health risk = no/unknown, Harm risk = no/unknown, Other risk = no/unknown.

**Table 3:** Initial and Final Logistic Regression Models Predicting Level of Risk (Low vs. High).

Key Characteristic	Initial Model					Final Model				
	*B*	*SE*	*p*	*OR*	95% CI	*B*	*SE*	*p*	*OR*	95% CI
Age	0.01	0.00	0.003	1.01	[1.00, 1.01]	0.01	0.00	0.001	1.01	[1.00, 1.01]
Sex	0.13	0.07	0.068	1.13	[0.99, 1.30]	0.13	0.07	0.053	1.14	[1.00, 1.31]
Ethnicity			0.130					-		
Asian	−0.18	0.18	0.319	0.84	[0.59, 1.19]	-	-	-	-	-
Mixed/other	0.16	0.29	0.579	1.17	[0.67, 2.06]	-	-	-	-	-
White	0.12	0.13	0.351	1.13	[0.88, 1.46]	-	-	-	-	-
Location missing from	0.34	0.09	<0.001	1.41	[1.17, 1.69]	0.34	0.09	<0.001	1.41	[1.18, 1.68]
Disability	0.22	0.11	0.050	1.24	[1.00, 1.55]	0.29	0.08	<0.001	1.33	[1.14, 1.58]
Mental illness	−0.13	0.11	0.254	0.88	[0.71, 1.09]	-	-	-	-	-
Absconder order	0.00	0.19	0.991	1.00	[0.69, 1.46]	-	-	-	-	-
Care order	−0.76	0.16	<0.001	0.47	[0.35, 0.63]	−0.76	0.15	<0.001	0.47	[0.35, 0.63]
Vulnerability risk	1.33	0.07	<0.001	3.78	[3.31, 4.32]	1.33	0.07	<0.001	3.77	[3.30, 4.31]
Personal circumstances risk	0.69	0.07	<0.001	1.98	[1.72, 2.29]	0.68	0.07	<0.001	1.97	[1.71, 2.28]
Health risk	1.19	0.08	<0.001	3.30	[2.82, 3.86]	1.23	0.08	<0.001	3.42	[2.94, 3.99]
Harm risk	0.51	0.07	<0.001	1.66	[1.45, 1.91]	0.52	0.07	<0.001	1.67	[1.46, 1.92]
Other risk	1.00	0.10	<0.001	2.72	[2.25, 3.28]	1.00	0.10	<0.001	2.72	[2.25, 3.28]

Note. Reference categories: Sex = male, Ethnicity = Black, Location missing from = other, Disability = no/unknown, Mental illness = no/unknown, Absconder order = no/unknown, Care order = no, Vulnerability risk = no/unknown, Personal circumstances risk = no/unknown, Health risk = no/unknown, Harm risk = no/unknown, Other risk = no/unknown.

**Table 4:** Initial and Final Logistic Regression Models Predicting Level of Risk (medium vs. high).

Key Characteristic	Initial Model					Final Model				
	*B*	*SE*	*p*	*OR*	95% CI	*B*	*SE*	*p*	*OR*	95% CI
Age	0.02	0.00	<0.001	1.02	[1.02, 1.03]	0.02	0.00	<0.001	1.02	[1.02, 1.03]
Sex	0.08	0.04	0.070	1.08	[0.99, 1.17]	0.08	0.04	0.047	1.09	[1.00, 1.18]
Ethnicity			0.094					-		
Asian	0.10	0.12	0.398	1.10	[0.88, 1.39]	-	-	-	-	-
Mixed/other	0.12	0.17	0.503	1.12	[0.80, 1.57]	-	-	-	-	-
White	0.19	0.08	0.019	1.21	[1.03, 1.43]	-	-	-	-	-
Location missing from	−0.02	0.05	0.730	0.98	[0.89, 1.09]	-	-	-	-	-
Disability	0.25	0.07	<0.001	1.28	[1.13, 1.45]	0.29	0.05	<0.001	1.33	[1.21, 1.46]
Mental illness	−0.07	0.06	0.298	0.94	[0.83, 1.06]	-	-	-	-	-
Absconder order	−0.29	0.10	0.003	0.75	[0.61, 0.91]	−0.30	0.10	0.002	0.74	[0.61, 0.90]
Care order	−0.77	0.10	<0.001	0.47	[0.38, 0.56]	−0.77	0.10	<0.001	0.46	[0.38, 0.56]
Vulnerability risk	−0.10	0.05	0.036	0.91	[0.83, 0.99]	−0.10	0.05	0.023	0.90	[0.83, 0.99]
Personal circumstances risk	0.44	0.05	<0.001	1.55	[1.41, 1.71]	0.44	0.05	<0.001	1.55	[1.42, 1.71]
Health risk	0.91	0.06	<0.001	2.49	[2.24, 2.78]	0.93	0.06	<0.001	2.54	[2.29, 2.83]
Harm risk	0.14	0.04	0.001	1.16	[1.06, 1.26]	0.15	0.04	<0.001	1.16	[1.07, 1.26]
Other risk	0.49	0.05	<0.001	1.63	[1.48, 1.79]	0.48	0.05	<0.001	1.62	[1.48, 1.78]

Note. Reference categories: Sex = male, Ethnicity = Black, Location missing from = other, Disability = no/unknown, Mental illness = no/unknown, Absconder order = no/unknown, Care order = no, Vulnerability risk = no/unknown, Personal circumstances risk = no/unknown, Health risk = no/unknown, Harm risk = no/unknown, Other risk = no/unknown.

## Data Availability

The data presented in this study are available on request from the corresponding author.

## Notes

1 The term ‘no apparent risk’ has now been revised to ‘very low risk’ to acknowledge that some degree of risk is always present, while clarifying that such a level does not warrant immediate police intervention.

2 Comparable demographic data from the 2021 England and Wales Census shows that the average age in England and Wales was 40 years, 49% of the general population were male and 51% female (the census did not include transgender as a response category, preventing comparison for this group). Regarding ethnicity, 82% were White, 4% were Black, 9% were Asian or Asian British individuals, and 5% were from Mixed, multiple, or other ethnic backgrounds. This confirms an over-representation of Black males in comparison to relevant national statistics.

## Abbreviations

The following abbreviations are used in this manuscript:

NCA	National Crime Agency
COMPACT	Community Policing and Case Tracking
PSV	Police Service Volunteer

## Appendix A

**Table A1:** Data Fields Requested from COMPACT.

Missing person details
Age (when reported missing)
Sex
Nationality
Ethnic appearance
Ethnicity
Marital status
Sexuality
Warning signals (PNC)
Disability and mental illness
(All fields)
Additional information
Absconder order type
Report details
Absent from (all)
Circumstances leading to disappearance
Person’s intentions when last seen?
Did the person prepare for an absence?
Document enquiries that the informant has made/intends to make
Is the person subject to a care order?
Detail the circumstances leading up to their disappearance
Risk assessment (Initial and Latest)
Person risk factor flags (person-specific, rather than case-specific)
Factors to be considered
Details
What do you consider to be the risk level?
State the reason for your choice of risk level
FOUND report
Did the misper go missing intentionally?
Specify reasons the misper gives, or the officer suspects, for going missing
Where was the misper found?
How far from the misper’s home address was he or she found?
What was the furthest distance travelled by the misper whilst they were missing?
How was the misper found?
Who found the misper?
Did the misper suffer any harm whilst missing?
What were the misper’s circumstances whilst they were missing?
How long was the misper missing for?
Full debrief

## Appendix B

**Table A2:** Deductive Coding Framework Groups for the 19 Initial Risk Factor Categories.

Group	Initial Risk Factor Categories
Vulnerability risk	IR1: Is the person vulnerable IR11: Subject to child protection IR13: Lacks ability to interact safely with others
Personal circumstances risk	IR2: Is the behaviour out of character IR5: Is there a reason for going missing IR6: Preparations for absence IR7: Did they fail to complete intentions IR17: Education, employment, or financial issue
Health risk	IR4: Likely to commit suicide IR10: Mental health issue IR14: Requires essential medication not available to them IR18: Drug or alcohol dependency
Harm risk	IR3: Likely to be subject of crime IR8: Family relationship problems or conflict/abuse IR9: Victim or perpetrator of domestic abuse IR12: Suffered or exposed to harm in previous missing incident IR15: Ongoing, bullying, harrassment, or community/cultural issues IR16: Involved in violent or racist incident
Other risk	IR19: Other factors influencing risk
